# A pilot study on efficacy and safety of a new salt substitute with very low sodium among hypertension patients on regular treatment

**DOI:** 10.1097/MD.0000000000019263

**Published:** 2020-02-21

**Authors:** Lihong Mu, Chenglong Li, Ting Liu, Wuxiang Xie, Ge Li, Meixian Wang, Ruoxi Wang, Huakun Rao, Qin He, Wen Wang, Yangfeng Wu

**Affiliations:** a Department of Epidemiology, School of Public Health and Management, Research Center for Medicine and Social Development, Innovation Center for Social Risk Governance in Health, Chongqing Medical University, Chongqing; b Peking University Clinical Research Institute, Peking University Health Science Center, Beijing; c Chongqing Nan’an District People's Hospital, Chongqing, China.

**Keywords:** blood pressure, hypertension, potassium, salt, sodium

## Abstract

**Objectives:**

To understand the possible effect of a novel salt substitute with very low sodium in reducing blood pressure, salt intake and use of anti–hypertensive medications among patients on regular medications, to inform the future randomized trials.

**Design:**

Single-arm pilot trial.

**Setting:**

A community health service center in Chongqing, China.

**Participants:**

A total of 43 patients with hypertension taking anti-hypertensive medications regularly.

**Intervention:**

Patients received the salt substitute with 18% sodium chloride for 8 weeks.

**Main outcome measures:**

Patients were followed up weekly for the use of antihypertensive medications and measurements of blood pressure. We collected 24-h urine before and after the trial to measure sodium and potassium intake.

**Results:**

Among 39 patients who completed the 8 weeks’ intervention, 30.8% patients stopped or reduced anti-hypertensive medications during the trial. For patients that stopped or reduced medication, the mean SBP and DBP before intervention were 122.1 ± 9.6 and 68.9 ± 9.4 mm Hg and both did not increase after intervention (SBP change: 2.8 mm Hg (−5.1, 10.8), *P* = .48; DBP change: 1.8 mm Hg (−2.2, 5.7), *P* = .38). For the rest patients, the mean SBP and DBP before intervention were 141.6 ± 16.9 and 74.6 ± 6.6 mm Hg but reduced significantly after the intervention (SBP change: −16.0 mm Hg (−21.3, −10.6), *P* < .001; DBP change: −5.5 mm Hg (−8.1, −2.9), *P* < .001). The 24-h urine sodium decreased (*P* < .001) and potassium increased (*P* < .001) among all patients. No severe adverse events were reported.

**Conclusions:**

The novel salt substitute showed potential in reducing blood pressure and use of antihypertensive medications. Further randomized double–blind controlled trial is warranted to validate these findings.

Clinical Trial Registration—URL:http://www.clinicaltrials.gov. Unique identifier: NCT03226327.

## Abstract 2

Supplemental Digital Content is available in the text

## 1 Introduction

As the most important risk factor for cardiovascular and cerebrovascular diseases, raised blood pressure is associated with the development of 47% coronary heart disease and 54% strokes.^[1]^ Furthermore, ∼50% of higher blood pressure–caused disease burden is associated with excessive salt intake.^[2]^ The World Health Organization recommends <5 g and Chinese Society of Nutrition recommends <6 g of salt per day for adults.^[3]^

Among salt reduction strategies, salt substitute was developed to reduce blood pressure by taking advantages of the blood pressure reduction effect from addition of potassium and at the same time reduction of sodium in the salt while keeping the taste of saltiness unchanged as much as possible. In the composition of salt substitute, usually 30% to 50% of sodium chloride is replaced by potassium chloride plus or not other minerals.^[4]^ There have been many high quality randomized controlled clinical trials demonstrating that salt substitute could effectively reduce blood pressure among patients with hypertension.^[5–9]^ However, such kind of salt substitutes is unable to reach the recommended level of <6 g/day^[10]^ for salt intake in Chinese population and may limit the effect of salt substitution to reach its highest possibility, since the current average salt intake is about 12 g per day in China.^[11]^

A novel salt substitute, named “Man Li Kang,” containing 18% of sodium chloride only, was developed to help people with difficulty in reduction of salt intake to reduce sodium intake by significantly a large amount so that the WHO recommended level of salt intake could be attained and its effect in lowering blood pressure can be maximized, without changing taste of food or habits of salt consumption.

In this study, we aimed to preliminarily understand the possible effect of the novel salt substitute in reducing blood pressure, salt intake and use of anti–hypertensive medications as well as its safety among hypertensive patients who are taking anti-hypertension medications regularly. The information gained from this study would inform the design for a randomized controlled trial to confirm the efficacy and safety of the novel product.

## 2 Methods

### 2.1 Study design

This pilot study was designed as a single–arm trial with 8 weeks of intervention using the novel salt substitute among hypertensive patients taking antihypertensive medications regularly.

### 2.2 Patient and public involvement

Patients and public were not involved in development of the research question or design of the study. They were also not involved in the recruitment to or conduct of the study. Besides, as an important and essential part of our procedure of obtaining patients and their family members’ written informed consent, sufficient time was ensured for them to give full consideration and assessment of the potential risks of the trial and burden of the intervention by themselves before signing written informed consent. After signing written informed consent, patients were assessed to verify whether they were eligible for inclusion and then entered into baseline investigation. General results, with all patients’ individual identifiable information (i.e., full name and other personal data) previously removed, of this trial will be available on reasonable demands.

### 2.3 Participants

From April to July in 2018, 69 participants were assessed for eligibility. After excluding 23 participants who did not meet the inclusion and exclusion criteria, a total of 43 patients with hypertension were recruited from a community health service center in Chongqing. To be included in the study, patients had to be diagnosed as hypertension and have taken antihypertensive medications without change in dose and types in past 3 months, and meet all of the following conditions:

1. aged ≥50 and ≤75 years;
2. not had plans to move out of the community in the next 3 months;
3. not cooking at home <3 times or 1 day during the study; and
4. provided written informed consent before enrollment in the trial.

Patients will be excluded from the study if they were having:

1. history of acute myocardial infarction or stroke in the past 3 months, history of malignancy or expected lifetime <1 year;
2. hypercortisolism or aldosteronism;
3. acute disease, such as upper respiratory infection, fever, and diarrhea;
4. salt substitute use in the family;
5. disease or disabilities that could exert potential influence on their adherence to the intervention, including deafness, dementia, as well as severe depression and other mental disorders;
6. family members not willing to use the salt substitute;
7. liver dysfunction;
8. anyone with abnormal serum potassium in family;
9. anyone using potassium-retaining diuretics in family.

The study has been approved by the Institutional Review Board of Peking University (IRB00001052−17110). All patients and their families had signed informed consent which illustrated the study's purpose, benefits and risks. The trial was registered at www.clinicaltrials.gov (NCT03226327).

### 2.4 Baseline

Subjects and their families were given informed consent and then completed baseline investigation at the clinic including blood pressure measurements, questionnaires gathering data of demography, lifestyle, history of diseases, and anti-hypertension medications, as well as collection of 24 h urinary samples.

### 2.5 Intervention

After baseline survey completed, research staff went to the patient's home and sealed all salt in each subject's household and replaced with the study salt substitute, Man Li Kang, which contained 18% of sodium chloride, 35% of potassium chloride, and 10% of calcium chloride. The study subjects were instructed to keep their habits of salt consumption unchanged. The intervention was implemented for 8 weeks for each patient.

### 2.6 Follow up

We conducted follow up once a week at study participant's home to measure blood pressure and collect data on the use of antihypertensive medications, and incidence of adverse events.

### 2.7 Blood pressure measurement

Sitting blood pressure, including systolic and diastolic blood pressure, was measured by trained staff in the clinic at baseline and patients’ home at each follow up visit, using electronic sphygmomanometer on the right arm, with appropriate cuff size. Patients were required to sit quietly for at least 5 min before the measurement and not to drink, eat, smoke, or carry out any physical activity in preceding 30 min.^[12]^ Blood pressure was measured three times at each visit with at least 1 min in between, and we used the average of three readings for data analysis.

### 2.8 Urinary measurements

At baseline and end of this trial, 24-h urinary samples were collected and sent to the laboratory at the First Affiliated Hospital of Chongqing Medical University for analysis. The 24 h urinary electrolyte, including sodium and potassium concentrations, were measured using HITACHI 7600 with the ion selective electrode method,^[13]^ while urine creatinine and urine microalbuminuria were measured with the pyrogallol red molybdenum method.^[14]^

### 2.9 Study outcomes

Primary outcomes were changes in systolic and diastolic blood pressure from baseline to the end of follow-up. Secondary outcomes included the change in hypertension control rate, namely proportion of patients with SBP < 140 and DBP < 90 mm Hg; the change in proportion of patients reaching Chinese Society of Nutrition recommended target for salt reduction, which was defined as the average daily sodium chloride intake as measured by 24 h urinary sodium below 6 g/day; and the proportion of patients with anti-hypertension medication reduction, which was defined as stopping or reducing the dose or number of anti-hypertension medications during the trial including both physician-agreed and self-decided reduction.

### 2.10 Statistical analysis

Our main analysis was based on per protocol data set, including a total of 39 patients who completed both baseline and follow-up assessments. Mean and standard deviation were used for description of continuous variables with normal distribution, median, and inter-quartile ranges for data of skewed distribution, and percentages for categorical variables. We assessed effects of intervention on SBP by using linear mixed model, with SBP levels as the dependent variable and the time of blood pressure measurement (visits: baseline or follow-up) as the independent variable included as random effect. To account for potential confounding effects, we adjusted for covariates including sex, age, BMI, and use of anti-hypertensive medication, fitted as fixed effects. We repeated the same analysis for DBP and pulse pressure (PP), and for patients with and without reduction of anti-hypertension medications.

For comparisons of urinary sodium and potassium excretion and the sodium-to-potassium ratio between baseline and end of intervention, linear mixed model was also used, with age, sex, and body mass index (BMI) adjusted.

In addition, we performed intention to treat analysis as sensitivity analysis, which included a total of 43 participants, with missing data imputed using the method of last observations carried forward.

All procedures of statistical analysis were performed using SAS version 9.4 software (SAS Institute, Cary, NC), with a two tailed *P* < .05 considered as statistically significant.

## 3 Results

We screened 69 participants for eligibility, among whom a total of 43 hypertensive patients meeting inclusion and exclusion criteria were enrolled, as was demonstrated in Patient Flow Diagram.

Baseline characteristics were presented for patients included in the study in Table 1. Overall, they aged 67.6 ± 7.9 years on average and had hypertension for almost 10 years and a mean BMI of 27.1 kg/m^2^. About half were women and half had a history of diabetes, coronary heart disease, or stroke.

**Table 1:**
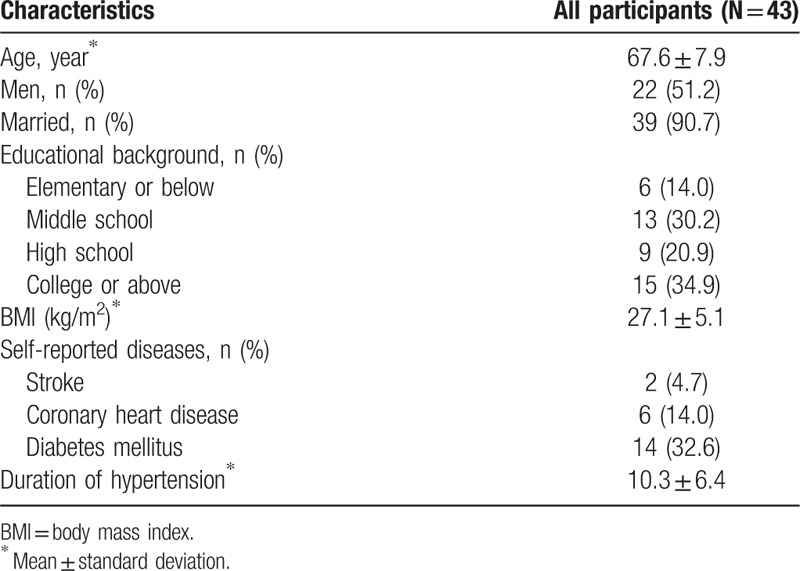
Baseline characteristics of study participants.

Tables 2–4 showed the mean baseline level and changes (95% CI) in systolic, diastolic, and PP during the trial for per protocol analysis, after adjusting for age, sex, BMI, and use of antihypertensive drugs using the linear mixed model. The mean changes in systolic, diastolic, and PP over 8 weeks was −11.7 mm Hg (−16.1 to −7.3 mm Hg, *P* < .001), −4.0 mm Hg (−6.2 to −1.9 mm Hg, *P* < .001), and −8.0 mm Hg (−11.3 to −4.7 mm Hg, *P* < .001), respectively. Meanwhile, significant reduction in SBP and PP appeared at the 1st week of intervention, while significant reduction in diastolic blood pressure appeared at the 2nd week of intervention. We did the same analysis for patients with and without reduction of antihypertension medications during the trial. Significant changes in SBP, DBP, and PP were only observed for patients who did not reduce medications. For patients who reduced medications, changes in SBP, DBP, and PP were not statistically significant. Interestingly, the baseline blood pressure level was lower for patients reducing than not reducing medications (122.1 mm Hg vs 141.6 mm Hg in SBP, 68.9 mm Hg vs 74.6 mm Hg in DBP, and 52.1 mm Hg vs 67.0 mm Hg in PP).

**Table 2:**
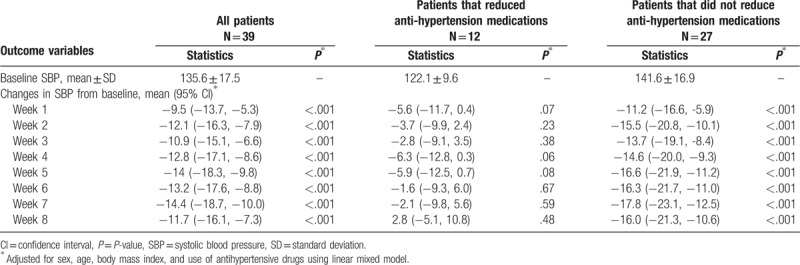
Baseline mean ± SD of SBP and changes in SBP from baseline during intervention, per protocol analysis.

**Table 3:**
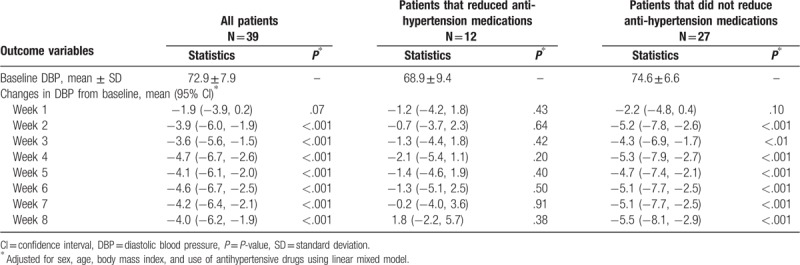
Baseline mean ± SD of DBP and changes in DBP from baseline during intervention, per protocol analysis.

**Table 4:**
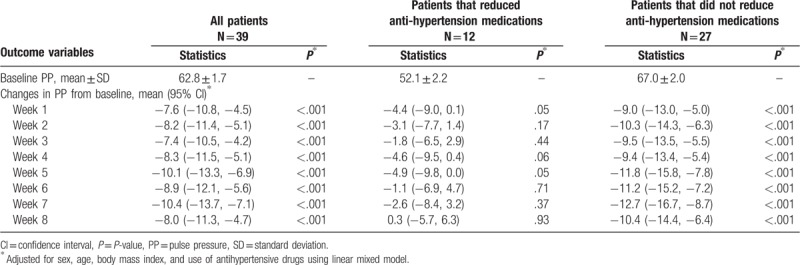
Baseline mean ± SD of pulse pressure (PP) and changes in PP from baseline during intervention, per protocol analysis.

**Figure 1:**
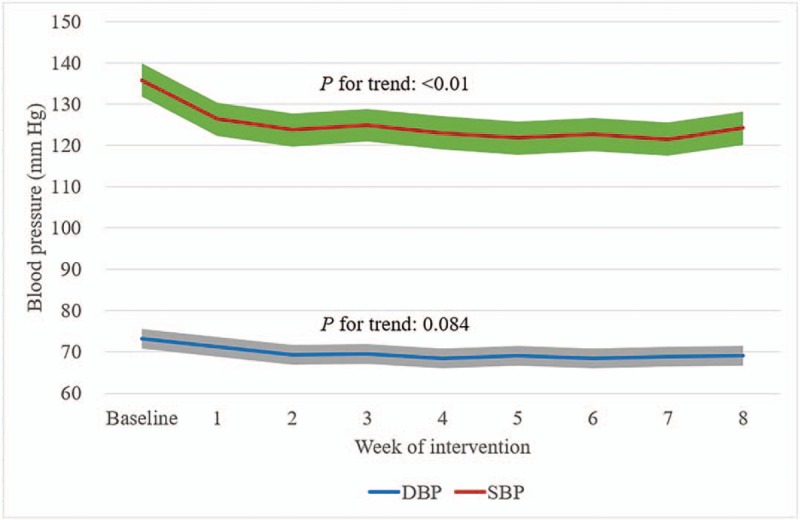
Trend over time of systolic and diastolic blood pressure for per protocol analysis, *P* values were calculated for the time effect using linear mixed models, after adjusting for sex, age, body mass index, and use of antihypertensive drugs. DBP = diastolic blood pressure, SBP = systolic blood pressure. Area of the shadow was indicative of the 95% confidence interval.

Similar results were observed among intention to treat population, −13.2 mm Hg reduction for SBP (−17.3 to −9.2 mm Hg, *P* < .001, Supplementary Table S1), −4.8 mm Hg for DBP (−6.8 to −2.8 mm Hg, *P* < .001, Supplementary Table S2), and −8.7 mm Hg for PP (−11.7 to −5.7 mm Hg, *P* < .001, Supplementary Table S3) at 8th week of intervention, respectively. We further analyzed changes in blood pressure according the type of anti-hypertensive medication, we did not find any significant differences in the changes in blood pressure between the groups (Supplementary Table S4). The trend over time in SBP was significant, after adjusting for sex, age, BMI, and use of antihypertensive medications, in both per protocol (Fig. 1) and intention to treat analysis (Supplementary Figure S1).

Table 5 showed the results of 24 h urinary tests. The daily sodium urinary excretion or salt intake estimated by urinary sodium decreased but not significantly after adjusting for potential cofounding variables. However, the potassium urinary excretion significantly increased by 8.8 mmol/day (1.5–16.1 mmol/day, *P* = .02) and the sodium-to-potassium ratio decreased significantly from baseline to end of trial (3.3–2.4, *P* < .001). Mean urinary creatinine and microalbuminuria did not change significantly.

**Table 5:**
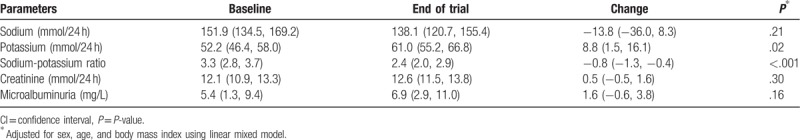
24 h urinary parameters, mean (95% CI)^∗^.

Proportion of patients with blood pressure controlled increased significantly from baseline to end of the trial (64–90%, *P* = .01), the increase in proportion with salt intake <6 g/day was not significant (23–28%, *P* = .56).

A total of 12 subjects have either stopped or reduced the dose or number of their anti-hypertension medications during intervention (Table 6), among which 4 patients had their physician's agreement for stopping or reducing medications. No serious adverse events were observed, with details shown in Supplementary Table S5.

**Table 6:**
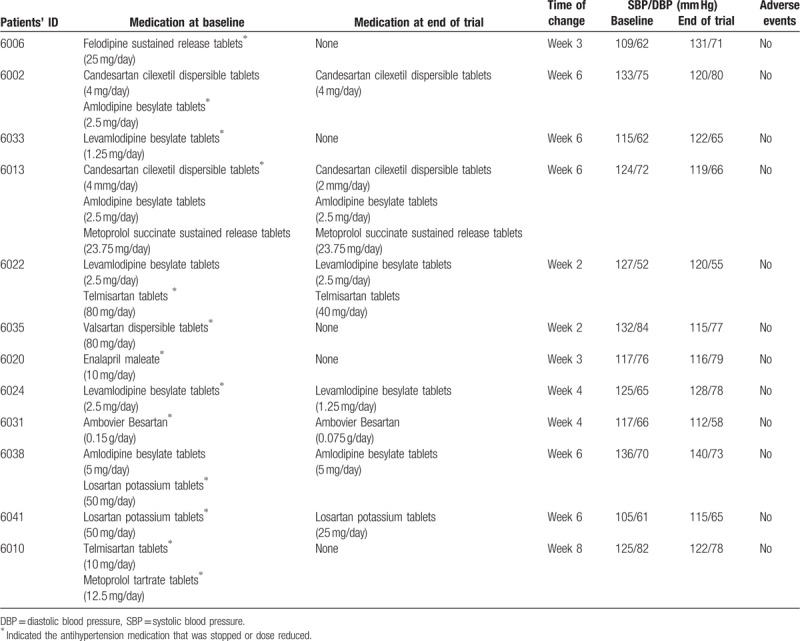
Details on drug reduction and withdrawal.

## 4 Discussion

This pilot study showed the possibility of reducing anti-hypertension medications by a novel salt substitute containing 18% of sodium chloride, in addition to its effect in achieving the reduction of blood pressure, among hypertensive patients who were taking anti-hypertension medications regularly. When confirmed, it will bear very important clinical as well as public health significance. Although the effect of salt substitute in reducing blood pressure has been proven by many well-conducted randomized trials,^[6]^ only few reported that salt substitute could effectively reduce the use of anti-hypertension medications.^[15]^ From both clinical and public health point of view, it offers an option additional to pharmaceutical and lifestyle change interventions for hypertension control and thus increases the population control of hypertension, which is currently about 15% only in China.^[16]^

Our observations that most of the patient who reduced medications had their blood pressure controlled fairly well already at baseline (mean SBP/DBP at 122/69 mm Hg) but decided by themselves to reduce medications during the trial indicated that the acceptability of the salt substitute for reducing blood pressure should be better than pharmaceutical treatments. That was the underlining reason why so many patients (over 30%) stopped or reduced their anti-hypertension medications during the trial when their blood pressure retuned to “normal” level by their own standards. In particular, mean blood pressure in these patients with reduction of medications did not increase during the trial, and there were four patients even reduced medications with agreement of their physicians. This phenomenon indicated that, with use of the study salt substitute, reduction of anti-hypertension medication use may be achievable for some patients without risking to increase their blood pressure. The observed contrast between patients who reduced and not reduced anti-hypertension medications in the effect of blood pressure reduction reinforced the effect of the study salt substitute on blood pressure lowering.

The above conclusion was also supported by our observations in changes of sodium and potassium intake estimated by 24 h urinary sodium and potassium excretion, where potassium intake increased significantly by 8.8 mmol/day (17%) and sodium to potassium ratio decreased significantly by −0.8. However, salt intake reduction, estimated by change in 24 h urinary sodium excretion, from baseline to end of trial and change in proportion of patients with salt intake meeting the Chinese Society of Nutrition's recommendation^[17]^ were both statistically insignificant. Communications with study patients at weekly visits revealed that some of them did not use completely the study salt substitute and might add regular salt during cooking. In fact, there were 4 patients who withdrew from the study because they did not like the taste of the study salt. Hence, the study salt needs further improvement in this aspect in order to increase its acceptability and long-term adherence.

In the term of adherence of the study salt substitute, previous studies reported an acceptable attitude among most subjects.^[18,19]^ Chun Huang^[20]^ mixed regular salt with low sodium salt substitute in ratios of 3:1, 1:1, and 1:3, respectively and applied these combinations into intervention of 1 to 4 weeks. With gradual increase in amount of low sodium salt substitute, study subjects adapted to the taste of the salt substitute step by step. In contrast, our study replaced the regular salt completely and suddenly for study subjects, without leaving sufficient time for patients to adapt to the study salt substitute's taste, which might help to partially explain the withdrawal of the four patients.

Nevertheless, the study also bears important limitations. First, it had no parallel control group and was not blinded due to its pilot nature, and thus the placebo effect could not be entirely ruled out. Second, due to its very small sample size, we were also unable to conclude on the safety of study salt substitute, despite that we did not receive any reports of severe adverse events. Third, we did not measure body weight at the end of the intervention, which prevents us from understanding the possible impact of changes in body weight on our results. However, previous studies showed that salt substitution should had no effect on body weight in such a short period.^[21]^ Nevertheless, the study successfully collected important information that could serve as basis for future randomized controlled trial to confirm our findings, with selecting study outcomes and estimation of study sample size in particular.

In conclusion, the novel salt substitute presented potential effects in blood pressure lowering and reducing use of antihypertensive medications, without reporting incidence of any serious adverse events. However, the substitute's taste and compliance required further improvement. And future randomized controlled trials were warranted to confirm our findings in the study salt substitute's effects of reducing blood pressure, anti-hypertension medication use and salt intake, as well as safety.

## Acknowledgments

We thank all of the participants and their families for their understanding and strong support of our work, all of the patient advisers for their efforts in collaboration with the study group and all of the members in the study group for their generous contributions to the study, and Health Source (Chongqing) Cardiovascular Health Technology for supplying the study salt substitute.

## Author contributions

**Data curation:** Chenglong Li.

**Formal analysis:** Chenglong Li.

**Funding acquisition:** Yangfeng Wu.

**Investigation:** Lihong Mu, Ting Liu, Ge Li, Meixian Wang, Ruoxi Wang, Huakun Rao, Qin He, Wen Wang.

**Methodology:** Yangfeng Wu, Chenglong Li, Wuxiang Xie.

**Project administration:** Lihong Mu, Ting Liu.

**Resources:** Yangfeng Wu, Lihong Mu, Ge Li.

**Software:** Wuxiang Xie.

**Supervision:** Yangfeng Wu.

**Writing – original draft:** Chenglong Li.

**Writing – review & editing:** Yangfeng Wu, Lihong Mu, Ting Liu, Wuxiang Xie, Ge Li, Meixian Wang, Ruoxi Wang, Huakun Rao, Qin He, Wen Wang.

## Supplementary Material

Supplemental Digital Content

## Supplementary Material

Supplemental Digital Content

## Supplementary Material

Supplemental Digital Content

## Supplementary Material

Supplemental Digital Content

## Supplementary Material

Supplemental Digital Content

## Supplementary Material

Supplemental Digital Content

## References

[1] Lawes CM Hoorn SV Rodgers A Global burden of blood–pressure–related disease. Lancet 2001;371:1513–8. 10.1016/S0140-6736(08)60655-8 18456100

[2] Asaria P Chisholm D Mathers C Chronic disease prevention: health effects and financial costs of strategies to reduce salt intake and control tobacco use. Lancet 2007;370:2044–53. 18063027 10.1016/S0140-6736(07)61698-5

[3] Yuan P Zhang SF Overview of excessive salt intake. Henan J Prev Med 2013;24:268–70.

[4] Chen X Ma JX Guo XL Domestic and foreign strategies and actions for reducing salt and preventing hypertension. Prev Med Tribune 2011;17:817–21.

[5] Hu JH Zhao LC Li X Effects of salt substitution on blood pressure using home measurements in essential hypertensive patients: a double–blinded randomized controlled trial. Chin J Hypertens 2014;22:42–6.

[6] Peng YG Li W Wen XX Effects of salt substitutes on blood pressure: a meta-analysis of randomized controlled trials. Am J Clin Nutr 2014;100:1448–54. 25411279 10.3945/ajcn.114.089235

[7] Adrian V Hernandez Erin E Systematic review and meta–analysis of the effects of low sodium salt substitutes on cardiovascular outcomes. J Am Coll Cardiol 2018;71:1749–1749.

[8] Feng JH Macgregor GA Can a low–sodium, high–potassium salt substitute reduce blood pressure in rural Chinese people? Nat Clin Pract Cardiovasc Med 2008;5:186–7. 18227813 10.1038/ncpcardio1122

[9] Hernandez AV Emonds EE Chen BA Effect of low-sodium salt substitutes on blood pressure, detected hypertension, stroke and mortality: a systematic review and meta-analysis of randomized controlled trials. Heart 2019;105:953–60. 30661034 10.1136/heartjnl-2018-314036

[10] Xiao X Liu Y Xiong X Observation of guiding effects by 24 h urinary sodium measurement on patients’ low sodium diet. Mod Hosp 2010;10:5–6.

[11] Zhou BF Stamler J Dennis B Nutrient intakes of middle-aged men and women in China, Japan, United Kingdom, and United States in the late 1990s: the INTERMAP study. J Hum Hypertens 2003;17:623–30. 13679952 10.1038/sj.jhh.1001605 PMC6561109

[12] Stamler J Chan Q Daviglus ML Relation of dietary sodium (salt) to blood pressure and its possible modulation by other dietary factors: the INTERMAP study. Hypertension 2018;71:631–7. 29507099 10.1161/HYPERTENSIONAHA.117.09928 PMC5843536

[13] Yin GS Zhang SL Yan L New index of using serum sodium and potassium and urine sodium and potassium jointly in screening primary aldosteronism in hypertensive patients. Natl Med J China 2010;90:962–6. 20646645

[14] Watanabe N Kamei S Ohkubo A Urinary protein as measured with a pyrogallol red-molybdate complex, manually and in a Hitachi 726 automated analyzer. Clin Chem 1986;32:1551–4. 3731450

[15] Zhou B Webster J Fu LY Intake of low sodium salt substitute for 3 years attenuates the increase in blood pressure in a rural population of North China—a randomized controlled trial. Int J Cardiol 2016;215:377–82. 27128565 10.1016/j.ijcard.2016.04.073

[16] Wu Y Huxley R Li L Prevalence, awareness, treatment, and control of hypertension in China: data from the China National Nutrition and Health Survey 2002. Circulation 2008;118:2679–86. 19106390 10.1161/CIRCULATIONAHA.108.788166

[17] Song P Li Y Jia S Status of salt intake among Chinese aged 60 and above in 2010–2012. J Hygiene Res 2016;45:714–7. 29903119

[18] Hu JH Preliminary Assessment of Blood Pressure Lowering Effects by Salt Substitution on Hypertensive Patients and Their Families, as well as Its Feasibility and Safety. Beijing, China: Peking Union Medical College Press; 2007.

[19] Zhang GH Field observation on the Effect of Blood Pressure with Low–Sodium/High–Potassium Salt Substitute and Health Education in Rural Community–Based Population of China. Shandong, China: Shandong University Press; 2012.

[20] Huang C Evaluation of the Short Term Effectiveness by High–Potassium Salt of Reducing Blood Pressure in a Nursing Home Population. Shanxi, China: Shanxi Medical University Press; 2013.

[21] Sarkkinen ES Kastarinen MJ Niskanen TH Feasibility and antihypertensive effect of replacing regular salt with mineral salt -rich in magnesium and potassium- in subjects with mildly elevated blood pressure. Nutr J 2011;10:88. 21888642 10.1186/1475-2891-10-88 PMC3175151

