# Supplementary material for: A pilot study on efficacy and safety of a new salt substitute with very low sodium among hypertension patients on regular treatment
Source: Medicine (Baltimore). 2020 Feb 21;99(8):e19263. doi: 10.1097/MD.0000000000019263 (PMC7034699; doi:10.1097/MD.0000000000019263)
Supplement: Supplemental Digital Content [file medi-99-e19263-s001.docx]

| Supplementary Table 1. Baseline mean ± SD of SBP and changes in SBP from baseline during intervention, intention to treat analysis with last observation carried forward imputation | | | | | | | | |
| --- | --- | --- | --- | --- | --- | --- | --- | --- |
| Outcome variables | All patients  N=43 | |  | Patients that reduced anti-hypertension medications  N=12 | |  | Patients that did not reduce anti-hypertension medications  N=31 | |
|  | Statistics | *P** |  | Statistics | *P** |  | Statistics | *P** |
| Baseline SBP, mean ± SD | 136.5 ±17.0 | - |  | 122.1 ±9.6 | - |  | 142.1 ±15.9 | - |
| Changes in SBP from baseline, mean (95% CI) * | | | | | | | | |
| Week 1 | -11.2 (-15.1, -7.3 ) | <0.001 |  | -5.6 (-11.7, 0.4 ) | 0.07 |  | -13.4 (-18.1, -8.6 ) | <0.001 |
| Week 2 | -13.5 (-17.4, -9.6 ) | <0.001 |  | -3.7 (-9.9, 2.4 ) | 0.23 |  | -17.0 (-21.7, -11.3 ) | <0.001 |
| Week 3 | -12.5 (-16.4, -8.5 ) | <0.001 |  | -2.8 (-9.1, 3.5 ) | 0.38 |  | -15.5 (-20.2, -10.8 ) | <0.001 |
| Week 4 | -14.2 (-18.2, -10.3 ) | <0.001 |  | -6.3 (-12.8, 0.3 ) | 0.06 |  | -16.3 (-21.0, -11.6 ) | <0.001 |
| Week 5 | -15.3 (-19.3, -11.4 ) | <0.001 |  | -5.9 (-12.5, 0.7 ) | 0.08 |  | -18.0 (-22.7, -13.2 ) | <0.001 |
| Week 6 | -14.6 (-18.6, -10.6 ) | <0.001 |  | -1.6 (-9.3, 6.0 ) | 0.67 |  | -17.8 (-22.5, -13.0 ) | <0.001 |
| Week 7 | -15.7 (-19.7, -11.6 ) | <0.001 |  | -2.1 (-9.8, 5.6 ) | 0.59 |  | -19.1 (-23.8, -14.3 ) | <0.001 |
| Week 8 | -13.2 (-17.3, -9.2 ) | <0.001 |  | 2.8 (-5.1, 10.8 ) | 0.48 |  | -17.4 (-22.2, -12.7 ) | <0.001 |

SBP: systolic blood pressure; SD: standard deviation; CI: confidence interval; P: P-value;

*Adjusted for sex, age, body mass index and use of antihypertensive drugs using linear mixed model.
