# Supplementary material for: A pilot study on efficacy and safety of a new salt substitute with very low sodium among hypertension patients on regular treatment
Source: Medicine (Baltimore). 2020 Feb 21;99(8):e19263. doi: 10.1097/MD.0000000000019263 (PMC7034699; doi:10.1097/MD.0000000000019263)
Supplement: Supplemental Digital Content [file medi-99-e19263-s002.docx]

| Supplementary Table 2. Baseline mean ± SD of DBP and changes in DBP from baseline during intervention, intention to treat analysis with last observation carried forward imputation | | | | | | | | |
| --- | --- | --- | --- | --- | --- | --- | --- | --- |
| Outcome variables | All patients  N=43 | |  | Patients that reduced anti-hypertension medications  N=12 | |  | Patients that did not reduce anti-hypertension medications  N=31 | |
|  | Statistics | *P** |  | Statistics | *P** |  | Statistics | *P** |
| Baseline DBP, mean ± SD | 73.8 ±8.5 | - |  | 68.9 ±9.4 | - |  | 75.7 ±7.5 | - |
| Changes in DBP from baseline, mean (95% CI) * | | | | | | | | |
| Week 1 | -3.0 (-4.9, -1.1 ) | <0.01 |  | -1.2 (-4.2, 1.8 ) | 0.43 |  | -3.7 (-6.0, -1.3 ) | <0.01 |
| Week 2 | -4.7 (-6.7, -2.8 ) | <0.001 |  | -0.7 (-3.7, 2.3 ) | 0.64 |  | -6.2 (-8.5, -3.8 ) | <0.001 |
| Week 3 | -4.4 (-6.4, -2.5 ) | <0.001 |  | -1.3 (-4.4, 1.8 ) | 0.42 |  | -5.4 (-7.7, -3.0 ) | <0.001 |
| Week 4 | -5.4 (-7.4, -3.5 ) | <0.001 |  | -2.1 (-5.4, 1.1 ) | 0.20 |  | -6.2 (-8.6, -3.9 ) | <0.001 |
| Week 5 | -4.9 (-6.8, -3.0 ) | <0.001 |  | -1.4 (-4.6, 1.9 ) | 0.40 |  | -5.8 (-8.1, -3.4 ) | <0.001 |
| Week 6 | -5.4 (-7.4, -3.4 ) | <0.001 |  | -1.3 (-5.1, 2.5 ) | 0.50 |  | -6.1 (-8.4, -3.7 ) | <0.001 |
| Week 7 | -5.0 (-7.0, -3.1 ) | <0.001 |  | -0.2 (-4.0, 3.6 ) | 0.91 |  | -6.1 (-8.4, -3.7 ) | <0.001 |
| Week 8 | -4.8 (-6.8, -2.8 ) | <0.001 |  | 1.8 (-2.2, 5.7 ) | 0.38 |  | -6.5 (-8.8, -4.1 ) | <0.001 |

DBP: diastolic blood pressure; SD: standard deviation; CI: confidence interval; P: P-value;

*Adjusted for sex, age, body mass index and use of antihypertensive drugs using linear mixed model.
