# Supplementary material for: A pilot study on efficacy and safety of a new salt substitute with very low sodium among hypertension patients on regular treatment
Source: Medicine (Baltimore). 2020 Feb 21;99(8):e19263. doi: 10.1097/MD.0000000000019263 (PMC7034699; doi:10.1097/MD.0000000000019263)
Supplement: Supplemental Digital Content [file medi-99-e19263-s003.docx]

| Supplementary Table 3. Baseline mean ± SD of PP and changes in PP from baseline during intervention, intention to treat analysis with last observation carried forward imputation | | | | | | | | |
| --- | --- | --- | --- | --- | --- | --- | --- | --- |
| Outcome variables | All patients  N=43 | |  | Patients that reduced anti-hypertension medications  N=12 | |  | Patients that did not reduce anti-hypertension medications  N=31 | |
|  | Statistics | *P** |  | Statistics | *P** |  | Statistics | *P** |
| Baseline PP, mean ± SD | 62.8 ±1.6 | - |  | 53.1 ±2.2 | - |  | 67.2 ±1.9 | - |
| Changes in PP from baseline, mean (95% CI) * | | | | | | | | |
| Week 1 | -8.2 (-11.1, -5.4 ) | <0.001 |  | -4.4 (-9.0, 0.1 ) | 0.06 |  | -9.7 (-13.2, -6.2 ) | <0.001 |
| Week 2 | -8.8 (-11.7, -6.0 ) | <0.001 |  | -3.5 (-8.0, 1.0 ) | 0.13 |  | -10.9 (-14.4, -7.3 ) | <0.001 |
| Week 3 | -8.1 (-11.0, -5.2 ) | <0.001 |  | -2.5 (-7.1, 2.0 ) | 0.27 |  | -10.1 (-13.7, -6.6 ) | <0.001 |
| Week 4 | -8.9 (-11.8, -6.0 ) | <0.001 |  | -5.6 (-10.2, -1.1 ) | <0.05 |  | -10.1 (-13.6, -6.5 ) | <0.001 |
| Week 5 | -10.6 (-13.5, -7.7 ) | <0.001 |  | -5.9 (-10.5, -1.4 ) | <0.05 |  | -12.2 (-15.7, -8.7 ) | <0.001 |
| Week 6 | -9.5 (-12.5, -6.5 ) | <0.001 |  | -3.0 (-7.6, 1.5 ) | 0.19 |  | -11.7 (-15.2, -8.2 ) | <0.001 |
| Week 7 | -10.9 (-13.8, -7.9 ) | <0.001 |  | -4.6 (-9.1, 0.0 ) | 0.05 |  | -13.0 (-16.5, -9.5 ) | <0.001 |
| Week 8 | -8.7 (-11.7, -5.7 ) | <0.001 |  | -1.8 (-6.4, 2.7 ) | 0.43 |  | -11.0 (-14.5, -7.5 ) | <0.001 |

PP: pulse pressure; SD: standard deviation; CI: confidence interval; P: P-value;

*Adjusted for sex, age, body mass index and use of antihypertensive drugs using linear mixed model.
