# Supplementary material for: A pilot study on efficacy and safety of a new salt substitute with very low sodium among hypertension patients on regular treatment
Source: Medicine (Baltimore). 2020 Feb 21;99(8):e19263. doi: 10.1097/MD.0000000000019263 (PMC7034699; doi:10.1097/MD.0000000000019263)
Supplement: Supplemental Digital Content [file medi-99-e19263-s004.docx]

Supplementary Table 4. Baseline and changes from baseline to end of trial in SBP, DBP and PP for patients on calcium channel blockers and ARB, per protocol analysis

| Blood pressure variables | Patients on ARBs  (N=12) | | |  | Patients on calcium channel blockers  (N=12) | | |  | Adjusted between group differences in changes * | |
| --- | --- | --- | --- | --- | --- | --- | --- | --- | --- | --- |
|  | Baseline (mean ± SD) | Change from baseline to end | *P* |  | Baseline (mean ± SD) | Change from baseline to end | *P* |  | Mean (95% CI) | *P* |
| SBP | 130.8 ±3.6 | -11.0 (-19.6, -2.5) | <0.05 |  | 137.9 ±3.6 | -11.9 (-20.4, -3.3) | <0.01 |  | -0.9 (-12.9, 11.2) | 0.89 |
| DBP | 73.0 ±2.1 | -4.6 (-8.5, -0.6) | <0.05 |  | 72.9 ±2.1 | -0.82 (-4.8, 3.1) | 0.68 |  | 3.8 (-1.8, 9.3) | 0.18 |
| PP | 57.8 ±3.0 | -6.4 (-12.9, 0.02) | 0.05 |  | 65.0 ±3.0 | -11.1 (-17.5, -4.6) | <0.001 |  | -4.6 (-13.7, 4.5) | 0.32 |

ARBs: Angiotensin Receptor Blockers; SBP: systolic blood pressure; DBP: diastolic blood pressure; PP: pulse pressure; SD: standard deviation; CI: confidence interval; P: P-value;

*Adjusted for sex, age, body mass index, use of antihypertensive drugs and anti-hypertensive medication type at baseline using linear mixed model.
