# Supplementary material for: A pilot study on efficacy and safety of a new salt substitute with very low sodium among hypertension patients on regular treatment
Source: Medicine (Baltimore). 2020 Feb 21;99(8):e19263. doi: 10.1097/MD.0000000000019263 (PMC7034699; doi:10.1097/MD.0000000000019263)
Supplement: Supplemental Digital Content [file medi-99-e19263-s005.docx]

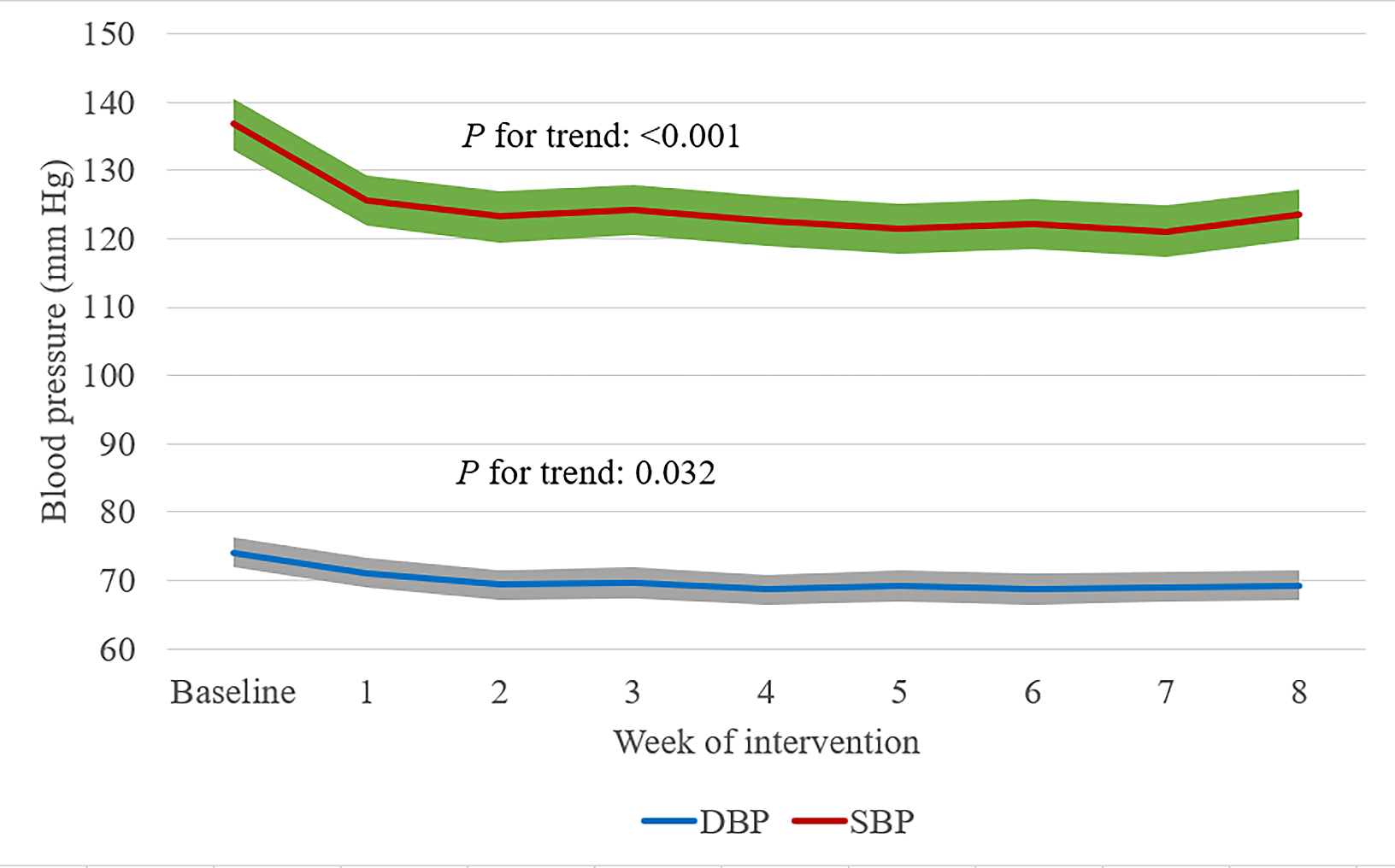


Supplementary Figure 1. Trend over time of systolic and diastolic blood pressure for intention to treat analysis, p values were calculated for the time effect using linear mixed models, after adjusting for sex, age, body mass index, and use of antihypertensive drugs. SBP: systolic blood pressure; DBP: diastolic blood pressure. Area of the shadow was indicative of the 95% confidence interval.
