# Supplementary material for: A pilot study on efficacy and safety of a new salt substitute with very low sodium among hypertension patients on regular treatment
Source: Medicine (Baltimore). 2020 Feb 21;99(8):e19263. doi: 10.1097/MD.0000000000019263 (PMC7034699; doi:10.1097/MD.0000000000019263)
Supplement: Supplemental Digital Content [file medi-99-e19263-s006.docx]

| Supplementary Table 5. Details of adverse events | |
| --- | --- |
| Adverse events | Incidence rate (%) |
|  |  |
| Dizziness | 7.7 |
| Debilitation | 5.1 |
| Headache | 2.6 |
| Neck pain | 2.6 |
| Cardialgia | 2.6 |
| Pruritus in neck, back and abdomen | 2.6 |
| Any adverse events | 15.4 |
